# Case Report of Acute Severe Hyponatremia Induced by Desmopressin Administration During Chemotherapy

**DOI:** 10.1002/ccr3.72192

**Published:** 2026-03-05

**Authors:** Koutaro Ono, Shugo Uematsu, Aya Yoshihara, Ayako Tsuboya, Shoichi Mori, Takashi Yoshioka, Shuichi Nawata

**Affiliations:** ^1^ Department of Hospital Pharmaceutics Showa Medical University School of Pharmacy Tokyo Japan; ^2^ Department of Pharmacy Showa Medical University Northern Yokohama Hospital Kanagawa Japan; ^3^ Respiratory Disease Center Showa Medical University Northern Yokohama Hospital Yokohama Japan; ^4^ Department of Internal Medicine Showa Medical University Northern Yokohama Hospital Yokohama Japan; ^5^ Institute of Clinical Epidemiology Showa Medical University Tokyo Japan; ^6^ Health Technology Assessment Unit, Department of Preventive Medicine and Public Health Keio University School of Medicine Tokyo Japan

**Keywords:** chemotherapy, cisplatin, desmopressin, hyponatremia, lung cancer, vinorelbine

## Abstract

Patients undergoing chemotherapy are prone to developing hyponatremia due to insufficient oral intake because of nausea and vomiting, administration of large volumes of intravenous fluids, syndrome of inappropriate antidiuretic hormone secretion induced by chemotherapeutic agents, adrenal insufficiency, and drug interactions. Severe hyponatremia during chemotherapy interferes with continuation of cancer treatment. In addition, it may be difficult to distinguish hyponatremia from chemotherapy‐related adverse effects because of symptom similarity. This report presented a case of desmopressin‐induced severe hyponatremia during chemotherapy and discussed the appropriateness of discontinuing desmopressin administration. A 70‐year‐old male was hospitalized for postoperative adjuvant chemotherapy with cisplatin and vinorelbine for a pathological stage IIB adenocarcinoma of the right lower lung lobes. The patient had been taking oral desmopressin (25 μg daily) for 4 years to treat nocturnal polyuria. Desmopressin was discontinued on day 1 of chemotherapy because of a contraindicated combination with dexamethasone, which was administered as an antiemetic. Desmopressin was resumed on day 6, but was followed by Common Terminology Criteria for Adverse Events (CTCAE) Grade 3 nausea, anorexia, and CTCAE Grade 4 hyponatremia (serum sodium concentration of 119 mEq/L) on day 7. Severe hyponatremia progressed following the resumption of desmopressin treatment. Desmopressin was not reintroduced thereafter, and subsequent chemotherapy cycles were continued without recurrence of hyponatremia. The patient completed all four planned adjuvant chemotherapy cycles. This case study highlighted the importance of carefully evaluating the necessity of continuing desmopressin administration and considering its discontinuation during chemotherapy to prevent the onset of hyponatremia.

## Key Clinical Message

Resuming desmopressin during chemotherapy can precipitate severe hyponatremia; its necessity should be reassessed and discontinuation considered to ensure safe continuation of cancer treatment.

## 1 Introduction

Hyponatremia is an important clinical condition that occurs during chemotherapy. It may be accompanied by severe symptoms such as nausea, vomiting, fatigue, and neurological disorders [1], which may necessitate chemotherapy discontinuation. Causes may include excessive fluid intake, poor oral intake, adrenal insufficiency, syndrome of inappropriate antidiuretic hormone secretion (SIADH), diabetes mellitus, electrolyte disturbances caused by cisplatin (CDDP), and drug interactions [1]. Although hyponatremia can be fatal in severe cases, its clinical manifestations are similar to chemotherapy‐associated symptoms, making it difficult to differentiate. Therefore, it is critical to monitor patient‐related factors and medications that may predispose patients to hyponatremia during chemotherapy and implement early detection and prompt intervention.

Desmopressin, which is widely used to treat diabetes insipidus and nocturnal polyuria, acts on vasopressin V2 receptors in the renal‐collecting ducts to enhance water reabsorption into the bloodstream and reduce urine output. However, it can also lead to intravascular fluid retention and dilutional hyponatremia and, therefore, requires careful monitoring [2], particularly during chemotherapy. In addition, dexamethasone, which is commonly used as an antiemetic agent during chemotherapy, is contraindicated when used concurrently with desmopressin [3]. This is likely due to dexamethasone‐induced fluid retention enhancing the antidiuretic effect of desmopressin and causing hyponatremia. Thus, dose adjustment of each medication and careful monitoring are essential [4, 5, 6]. When administering agents such as desmopressin that may induce hyponatremia, careful consideration of treatment necessity and appropriate timing of reinitiation are critical.

This report presents a case wherein desmopressin was discontinued during chemotherapy because of an elevated risk of hyponatremia.

## 2 Case History and Examination

A 70‐year‐old male underwent a robot‐assisted right lower lobectomy for clinical stage IB (cT2aN0M0) adenocarcinoma of the right lower lobe and was diagnosed with pathological stage IIB (pT3N0M0) adenocarcinoma. Two months later, the patient was hospitalized for adjuvant chemotherapy with CDDP and vinorelbine (VNR). The medical history of the patient included nocturnal polyuria, type 2 diabetes mellitus, myocardial infarction, bronchial asthma, benign prostatic hyperplasia, and hypertension. To manage nocturnal polyuria, the patient had been taking oral desmopressin (25 μg daily) for the previous 4 years. The patient was also receiving mirabegron, linagliptin, pioglitazone, azilsartan, mecobalamin, a combination tablet of low‐dose aspirin and lansoprazole, rebamipide, omega‐3‐acid ethyl esters, and magnesium oxide to treat other comorbidities. The patient had a smoking history of 20 cigarettes per day for 30 years, no history of alcohol consumption, and no drug allergies. The height and weight of the patient were 171 cm and 75 kg, respectively. Blood and biochemical test results were as follows: white blood cell count 6160/μL, hemoglobin 15.1 g/dL, platelets 217,000/μL, neutrophil count 4040/μL, blood urea nitrogen 14.0 mg/dL, creatinine 0.75 mg/dL, estimated glomerular filtration rate 78.5 mL/min/1.73 m^2^, serum sodium (Na) concentration 137 mEq/L, serum chlorine (Cl) concentration 103 mEq/L, serum potassium (K) concentration 4.2 mEq/L, total bilirubin 1.2 mg/dL, aspartate aminotransferase 34 mg/dL, alanine aminotransferase 45 mg/dL, C‐reactive protein 2.69 mg/dL, and blood glucose 121 mg/dL.

## 3 Differential Diagnosis, Investigations, and Treatment

**FIGURE 1:**
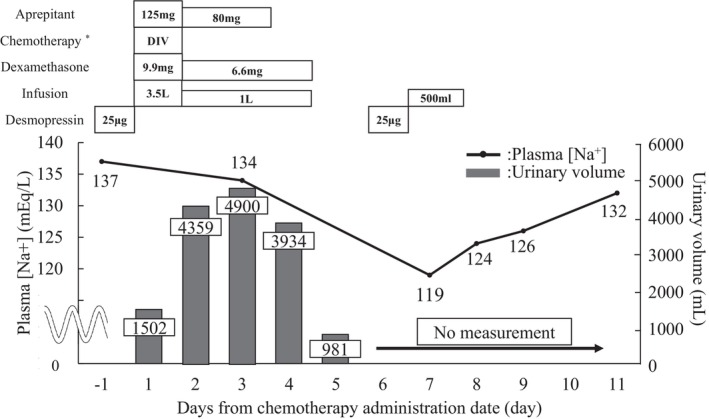
Serum sodium concentration in first cycle. Changes in plasma sodium concentration and urinary volume during the first chemotherapy cycle and timing of concomitant medication administration *Cisplatin 80 mg/m^2^, vinorelbine 25 mg/m^2^, infusion 3 L, Mg 10 mEq, furosemide 20 mg, palonosetron 0.75 mg, and dexamethasone 9.9 mg.

Adjuvant chemotherapy with CDDP plus VNR was administered according to our institutional chemotherapy schedule (Data S1). The patient received antiemetic therapy for highly emetogenic chemotherapy and hydration therapy to prevent CDDP‐induced nephrotoxicity, with CDDP administered at 80 mg/m^2^ on day 1 and VNR administered at 25 mg/m^2^ on days 1 and 8. At chemotherapy, the patient received 3500 mL of intravenous fluids, and 20 mg of furosemide was administered, followed by 1000 mL per day for the next 3 d, and oral fluid intake of 1.5–2.5 L per day. Although urine output of approximately 4000 mL/day was maintained for 3 day post‐chemotherapy, the overall fluid balance was considered positive. Oral desmopressin was discontinued from day 1 of chemotherapy as dexamethasone is contraindicated. Following chemotherapy initiation, the patient developed Common Terminology Criteria for Adverse Events (CTCAE) Grade 2 nausea, which improved following administration of 5 mg oral metoclopramide; however, oral intake by the patient remained low. The patient developed CTCAE Grade 3 nausea and a decreased appetite on day 7 after resuming desmopressin administration. Blood tests revealed a serum Na concentration of 119 mEq/L, corresponding to CTCAE Grade 4 hyponatremia (Figure 1). VNR administration was discontinued on day 8. No abnormalities were detected in the other electrolyte concentrations and no renal dysfunction was observed (Table 1). The urinary Na concentration increased to 40.0 mEq/L. No increase in antidiuretic hormone secretion or decrease in adrenocorticotropic hormone or cortisol concentration was observed; therefore, we eliminated SIADH and adrenal insufficiency.

**TABLE 1:** Laboratory test results.

Date	Unit	Before Chemotherapy	3	7	8	9	11
Body temperature	°C	36.3	36.8	36.4	36.4	36.4	36.3
Heart rate	bpm	69	85	76	79	78	69
Blood pressure	mmHg	124/86	145/83	133/98	108/70	111/76	126/78
Respiratory rate	/min	18	16	16	17	17	18
WBC	/μL	6160	9260	5310	3930	5010	
Hb	g/dL	15.1	15.3	15.9	16.9	16.3	
Plt	/μL	217,000	166,000	149,000	196,000	235,000	
Neutophil	/μL	4040	8450	3880	2740	3260	
Alb	/μL	4.2	3.9	3.5	3.7	3.8	
Creatinine	mg/dL	0.75	0.86	0.75	0.79	0.9	0.8
eGFR	mL/min/1.73 m^2^	78.5	67.6	78.5	74.2	64.3	73.2
BUN	mg/dL	14	18.6	18.7	14.9	16.9	9.0
Serum Na	mEq/L	137	134	119	124	126	132
Serum Cl	mEq/L	103	98	85	90	91	97
Serum K	mEq/L	4.2	4.5	4.5	4.5	4.9	43
Total bilirubin	mg/dL	1.2	1.2	1.3	1.2	0.9	
AST	mg/dL	34	35	29	34	32	
ALT	mg/dL	45	49	58	63	66	
CRP	mg/dL	2.69	0.39	1.16	0.94	0.5	
Blood glucose	mg/dL	121	169	104	147	109	
Urine specific gravity				1.005		1.012	
Urine creatinine	mg/dL			0.1		1.0	
Urine Na	mEq/L			40		18	
Urine Cl	mEq/L			32		14	
Urine K	mEq/L			5.7		16.9	
Urine osmolality	mOSM/kg H_2_O			167		368	
Serum osmolality	mOSM/kg			260			270
TSH	μIU/mL				1.01		
FT3	pg/mL				2.35		
FT4	ng/dL				1.91		
Renin activity	ng/mL/h				11.1		
Adrenocorticotropic hormone	pg/mL				26.1		
Cortisol	μg/dL				18		
ADH	pg/mL				0.8		

Abbreviations: ADH, antidiuretic hormone; Alb, serum albumin; ALT, alanine aminotransferase; AST, aspartate aminotransferase; BUN, blood urea nitrogen; Cl, chlorine concentration; CRP, C‐reactive protein; eGFR, estimated GFR; FT3, free triiodothyronine; FT4, free thyroxine; Hb, hemoglobin; K, potassium concentration; Na, sodium concentration; Plt, platelets; T‐Bil, total serum bilirubin; TSH, thyroid‐stimulating hormone; WBC, white blood cell count.

## 4 Outcome and Follow‐Up

**FIGURE 2:**
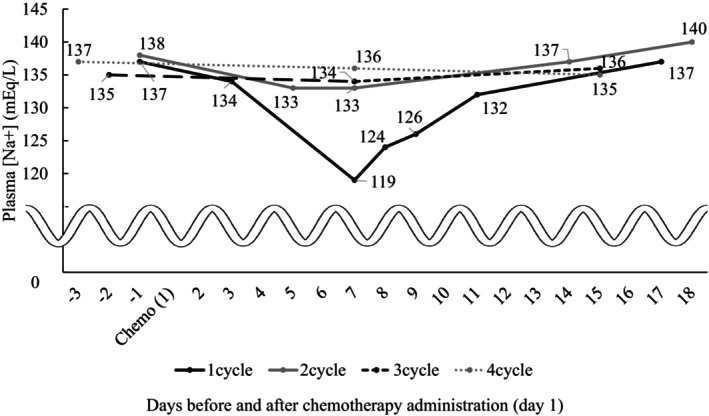
Serum sodium concentration for each cycle. Changes in plasma sodium concentration pre‐ and post‐chemotherapy across four treatment cycles.

Following the discontinuation of oral administration of desmopressin, administration of 500 mL intravenous normal saline and restriction of oral fluid intake, the serum Na concentration of the patient gradually improved and nausea was resolved. Desmopressin was not resumed thereafter, and the patient continued the same chemotherapy regimen of CDDP plus VNR without hyponatremia recurrence during subsequent cycles (Figure 2).

## 5 Discussion

Hyponatremia incidence associated with oral desmopressin was higher in the first 12 weeks of treatment. Among patients with a baseline serum Na concentration ≥ 140 mEq/L, hyponatremia occurs in approximately 5.1%. In contrast, those with lower baseline concentration (135–139 mEq/L) experience an incidence as high as 14.9% [7]. Since hyponatremia incidence (serum Na concentration < 135 mEq/L) among patients with cancer is approximately 20%–40%, the risk of desmopressin‐induced hyponatremia is considered high [8, 9]. Additionally, the effect of chemotherapy differs depending on the agents used; hyponatremia incidence is reported to be 11.9% with platinum‐based agents and 3.8% with non‐platinum agents. In the present case, no hyponatremia occurred following the discontinuation of oral desmopressin from the second cycle onward. Collectively, oral desmopressin may be a major contributor to the development of severe hyponatremia in this patient. However, as this patient had been taking oral desmopressin for a long period, the risk of hyponatremia due to desmopressin monotherapy was considered low. Therefore, severe hyponatremia was caused by reduced serum Na concentration, due to multiple factors (e.g., reduced Na intake, fluid overload, and enhanced urinary Na excretion due to furosemide administration), along with desmopressin‐induced fluid retention.

Patients with cancer are prone to developing hyponatremia [10], and SIADH accounts for a significant proportion of these cases. It is also well‐established that CDDP carries a high risk of hyponatremia through either renal salt‐wasting syndrome (RSWS) or SIADH [11, 12]. This patient was initially suspected of having drug‐induced SIADH or RSWS, as we found elevated urinary Na excretion, a decrease in serum osmolality, and an increase in urinary osmolality. However, the acute onset of symptoms following desmopressin administration, rapid improvement of symptoms after desmopressin discontinuation, and the absence of symptoms after the second cycle of chemotherapy during desmopressin interruption suggested that CDDP and VNR were not the causes. Furosemide‐induced increases in Na excretion were thought to be the most likely cause. Cisplatin‐induced RSWS was also evaluated; however, the decrease in urine Na levels 2 days after the onset of hyponatremia, as well as the absence of hyponatremia during the second and subsequent cycles of treatment, indicate that cisplatin's involvement was small.

However, oral desmopressin was discontinued on day 1 of chemotherapy because the concomitant use of dexamethasone, an antiemetic agent, is contraindicated. Considering the half‐life of desmopressin (approximately 2.5 h), a sufficient interval was allowed before chemotherapy initiation. Additionly, no decrease in serum Na concentration was observed on day 3, suggesting that no interaction with dexamethasone had occurred. Although the biological half‐life of dexamethasone (approximately 36–54 h) may have allowed its effects to persist at desmopressin resumption, the cortisol concentration was not suppressed when hyponatremia developed, indicating that residual effects of dexamethasone were unlikely. Thus, hyponatremia in this case was not caused by an interaction between desmopressin and dexamethasone. However, owing to this interaction, we could not eliminate the possibility that severe hyponatremia could develop early if desmopressin administration was continued during chemotherapy. Therefore, waiting at least 1 week before continuing desmopressin administration is necessary for avoiding any adverse effects.

## 6 Conclusions

Patients undergoing chemotherapy are at risk of developing hyponatremia owing to the side effects of anticancer drugs. As in the present case, desmopressin to treat nocturnal polyuria should not be administered under potential hyponatremic conditions. When administering anticancer drugs, it is advisable to assess the necessity of continuing hyponatremia‐prone agents, such as desmopressin, and temporarily suspend their administration during the anticancer drug administration period. Additionally, the decision to resume desmopressin administration should be made after considering the potential interactions with anticancer agents and dexamethasone, ensuring an interval of approximately 1 week between dexamethasone administration and the resumption of desmopressin administration.

## Author Contributions

**Koutaro Ono:** conceptualization, data curation, formal analysis, investigation, methodology, project administration, resources, supervision, writing – original draft, writing – review and editing. **Shugo Uematsu:** conceptualization, data curation, formal analysis, investigation, methodology, project administration, resources, supervision, writing – original draft, writing – review and editing. **Aya Yoshihara:** conceptualization, data curation, formal analysis, investigation, methodology, project administration, resources, supervision, writing – original draft, writing – review and editing. **Ayako Tsuboya:** conceptualization, formal analysis, investigation, methodology, project administration, resources, writing – original draft, writing – review and editing. **Shoichi Mori:** conceptualization, resources. **Takashi Yoshioka:** conceptualization, data curation, methodology, project administration, resources, supervision, writing – original draft, writing – review and editing. **Shuichi Nawata:** formal analysis, project administration, writing – original draft, writing – review and editing.

## Funding

The authors have nothing to report.

## Ethics Statement

The authors have nothing to report.

## Consent

Written informed consent was obtained from the patient. All specific patient information was obtained.

## Conflicts of Interest

The authors declare no conflicts of interest.

## Supporting information


**Data S1:** Cisplatin plus vinorelbine regimen schedule.

## Data Availability

The authors have nothing to report.
