# Supplementary material for: Case Report of Acute Severe Hyponatremia Induced by Desmopressin Administration During Chemotherapy
Source: Clin Case Rep. 2026 Mar 5;14(3):e72192. doi: 10.1002/ccr3.72192 (PMC12962875; doi:10.1002/ccr3.72192)
Supplement: Supplementary file 1 — Data S1: Cisplatin plus vinorelbine regimen schedule. [file CCR3-14-e72192-s001.docx]

Additional file 1.

Cisplatin + vinorelbine

Day 1

1. Renger’s solution 500 mL for 1.5 h

2. Magnesium Sulfate Hydrate (10 mEq) 10 mL ＋ Saline 500 mL for 1.5 h

3. Palonosetron 50 mL ＋ dexamethasone (9.9 mg) for 15 min

4. Vinorelbine (25 mg/m^2^) ＋ saline 50 mL for 6 min

5. Saline 50 mL

6. Cisplatin (80 mg/m^2^) 150 mL ＋ saline 300 mL for 2 h

7. Renger’s solution 1000 mL for 3 h

[Bypass injection] furosemide (20 mg )＋ saline 50 mL for 15 min

8. Maintenance infusion of 500 mL for 1.5 h

9. Renger’s solution 500 mL for 1.5 h

Days 2–4

1. Renger’s solution 500 mL for 2 h
2. Maintenance infusion 500 mL + dexamethasone (6.6 mg) for 2 h
